# Tardive Dyskinesia Following Low-Dose Risperidone

**DOI:** 10.7759/cureus.32319

**Published:** 2022-12-08

**Authors:** Ahmad E Rokon, Faisal A Alsomali, Malek A Alrasheed, Abdulrahman D Alharbi, Moustafa S Alhamadh, Abdulmajeed M Alqahtani, Farah K Alhamidy, Meshal R Alotaibi

**Affiliations:** 1 College of Medicine, King Saud Bin Abdulaziz University for Health Sciences, Riyadh, SAU; 2 Ministry of the National Guard-Health Affairs, King Abdullah International Medical Research Center, Riyadh, SAU; 3 Adult Mental Health, King Abdulaziz Medical City, Riyadh, SAU

**Keywords:** movement disorder, neurology, extrapyramidal symptoms, tardive dyskinesia, psychosis

## Abstract

Tardive dyskinesia is an involuntary athetoid or choreiform movement lasting a minimum of a few weeks. It is associated with the use of neuroleptic medication for at least three months and persists beyond four to eight weeks. Tardive dyskinesia usually occurs as a result of the long-term use of dopamine receptor-blocking agents, mainly first-generation antipsychotics or a high-dose, second-generation antipsychotic.

We present a case of a 28-year-old female with osteogenesis imperfecta presented later with major depressive disorder with psychotic features. She was given a low-dose second-generation antipsychotic, namely, risperidone (2 mg) for psychosis for a cumulative duration of three months. As a result, she developed extrapyramidal symptoms in the form of akathisia, axial dystonia, involuntary movement of the right hand, and smacking movement of the lips. Symptoms persisted for more than eight weeks despite discontinuing risperidone and switching to quetiapine. After the exclusion of other differential diagnoses, she was labeled as a case of tardive dyskinesia. More studies are needed to assess whether undiscovered contributing factors to tardive dyskinesia exist and to understand how second-generation antipsychotics (SGAs) contribute to the development of tardive dyskinesia.

## Introduction

Tardive dyskinesia (TD) is a constellation of abnormal involuntary stereotyped movements attributed to months or years of treatment with antipsychotic agents [1,2].

According to the Diagnostic and Statistical Manual of Mental Disorders, Fifth Edition (DSM-5), TD is an involuntary athetoid or choreiform movement that lasts a minimum of a few weeks and is associated with the use of a neuroleptic medication for at least three months and persists beyond four to eight weeks [3].

Psychosis is a syndrome that includes hallucinations, delusions, disorganized thought, speech, or behavior [3]. It is typically treated with antipsychotics, which have two types: first-generation antipsychotics (FGAs) and second-generation antipsychotics (SGAs) [3]. FGAs are dopamine receptor antagonists, which are also known as typical antipsychotics, while SGAs are serotonin-dopamine antagonists, which are also known as atypical antipsychotics [4,5]. An example of SGAs is risperidone. SGAs are known to have a lower risk of inducing extrapyramidal symptoms (EPS) and TD compared to FGAs. However, there is a variation between the medication in the same class as risperidone associated with a higher risk of developing TD, And at a high dose, the difference in the risk of inducing TD between FGAs and SGAs is negligible. But TD is rarely reported with low-dose monotherapy of SGAs [6].

Moreover, individuals over 45 are more likely to have TD compared to young adults and adults [7]. Adding these facts together, TD is less likely to develop in a young patient after using a low dose of SGAs. However, we present a case of a 28-year-old female diagnosed with TD after using 2 mg of SGAs for a cumulative period of 3 months with symptoms that last beyond eight weeks.

## Case presentation

A 28-year-old female, known case of osteogenesis imperfecta (OI), presented to the clinic complaining of a first episode of major depressive disorder (MDD) with symptoms including depressed mood, loss of interest, loss of appetite, insomnia, and weight loss. Along with aggression, persecutory delusions thinking her family wants to hurt her and disorganized thoughts in the form of loose association and echolalia were present. The Initial impression was MDD with psychotic features, and she was given citalopram and risperidone, both were increased gradually to reach 20 mg and 2 mg, respectively.

The patient was non-compliant and continued on the plan for only two months, after which she stopped taking any medications and stayed off medications for around two months. During this time, her psychosis became worse, as she started to hurt herself and threatened to hurt her family. She also stopped eating with them, thinking they would poison her, but there were no movement abnormalities during this time. After being off medications for two months, she visited the mental health clinic, and the plan was to resume the 2 mg risperidone.

After four weeks, she came to the emergency department with EPS in the form of akathisia, axial dystonia, involuntary movement of the right hand, and smacking movement of the lips. The plan was to discontinue risperidone gradually, and she was discharged. After six weeks, she was referred from mental health to neurology for assessment, as she was still having the same symptoms. On examination, the patient showed involuntary head-turning movement while walking, trunk tilting movement to the right, involuntary shoulder adduction and elbow flexion movement, lip smacking and akathisia while sitting without parkinsonian features (tremor, cogwheel rigidity, or bradykinesia). The plan was to schedule a follow-up after two weeks to reassess again and to consider starting a low-potency antipsychotic, namely, quetiapine. After two weeks, her symptoms did not subside, and two consultants confirmed the diagnosis of TD and prescribed her quetiapine (12.5 mg) after excluding other differential diagnoses by careful history, complete neurological examination, laboratory investigations, and imaging.

After four months, she returned to the clinic, as her symptoms did not improve. Her father stated that she stopped taking her medication one month ago because she was delusional that her family wanted to hurt her with the medication, which worsened her TD and psychosis. Quetiapine was started, and she was scheduled for regular follow-ups.

On further visits, the patient’s condition didn’t show a significant improvement, and she was offered multiple medications and treatment plans. Still, she was non-compliant, which made it harder to assess the effect of the medications on treating her TD. The patient continued to follow up with neurology and mental health for around one year until now, as her TD showed no improvement with worsening of psychosis, resulting in multiple emergency room (ER) visits for psychotic episodes.

Tests and investigations

Apart from a positive COL1A1 mutation, her complete blood count, liver function test, inflammatory markers, autoimmune antibodies, renal function test, and head MRI were unremarkable.

## Discussion

It is uncommon to develop TD with a low dose of SGAs in young adults. In the following article, we discussed a patient with OI and MDD with psychosis who was later diagnosed with TD as a result of using 2 mg risperidone for a cumulative period of three months with symptoms that lasted beyond eight weeks in a 28-year-old female.

There are several established risk factors for TD, including advanced age, female gender, minimum exposure of three months to antipsychotics, high dosage of antipsychotics, and co-administration of antipsychotics with antiparkinsonian agents [8,9]. Although our patient had two risk factors at the time of TD diagnosis - female gender and minimum exposure of three months to antipsychotics, she was using a low dose of SGAs, specifically, 2 mg of risperidone, which is less likely to cause motor abnormalities, such as TD, as it usually happened with a dosage of 6 mg or more [10]. Moreover, studies showed that a dose of 6 mg risperidone might cause unbalance in the 5HT2/D2 blocking effect, resulting in more affinity for D2 receptors, which produce comparable EPS with FGAs [11]. This raises the question of whether other unstudied contributing factors are involved, including the severity of psychosis or concomitant chronic or autoimmune disease.

The medication used in our patient is risperidone, which is an SGA. The introduction of SGAs a decade ago anticipated decreasing the risk of acute EPS and TD because these drugs have a weaker affinity for blocking the dopamine receptor. However, evidence from prospective and retrospective studies comparing FGAs and SGAs showed that the difference between these medication classes in inducing acute EPS and TD is less than initially thought, especially if SGAs were given in high doses [12,13]. Over the past three to four years, the number of TD cases associated with risperidone or olanzapine has risen. SGAs have drawn more and more attention to TD. This may be due to more people receiving these prescriptions today and for longer durations. However, it has been rarely reported that monotherapy with low-dose SGAs causes TD.

By carefully reviewing 12 cases of risperidone-induced TD, seven cases showed TD after using 6 mg or more of risperidone [14-19], three cases showed TD with less than 6 mg of risperidone, but with previous exposure to or concomitant use of FGAs [19-21], one case didn’t report the dosage of risperidone [22] and only one case of a 21-year-old-female developed TD after five months on monotherapy of 1.5 mg risperidone [23]. What makes the mechanism behind SGAs causing TD difficult to understand is that one of the reported cases showed that a reduction in the risperidone dose to 2 mg from 6 mg completely treated the patient [18], which is the same dose that caused TD in our patient and almost the same as the dose of the previously reported case. More studies are needed to understand how SGAs contribute to the development of TD, as this will help reduce its risk.

## Conclusions

We believe that our case report will add valuable data to the existing literature, as this is the second case where monotherapy treatment with risperidone caused TD in a dose of less than 6 mg. More studies are needed to assess whether undiscovered contributing factors to TD exist and to understand how SGAs contribute to the development of TD. We recommend that physician should be more aware and continue regular follow-ups while prescribing SGAs even at a small dose.
